# Automatically Diagnosing Disk Bulge and Disk Herniation With Lumbar Magnetic Resonance Images by Using Deep Convolutional Neural Networks: Method Development Study

**DOI:** 10.2196/14755

**Published:** 2021-05-21

**Authors:** Qiong Pan, Kai Zhang, Lin He, Zhou Dong, Lei Zhang, Xiaohang Wu, Yi Wu, Yanjun Gao

**Affiliations:** 1 School of Telecommunications Engineering Xidian University Xi’an China; 2 College of Science Northwest A&F University Yangling China; 3 School of Computer Science and Technology Xidian University Xi'an China; 4 SenseTime Group Limited Shanghai China; 5 School of Computer Science Northwestern Polytechnical University Xi'an China; 6 State Key Laboratory of Ophthalmology Zhongshan Ophthalmic Center Sun Yat-sen University Guangzhou China; 7 Medical Imaging Department The Affiliated Hospital of Northwest University Xi’an Number 3 Hospital Xi’an China; 8 Xi’an Key Laboratory of Cardiovascular and Cerebrovascular Diseases The Affiliated Hospital of Northwest University Xi’an Number 3 Hospital Xi'an China

**Keywords:** deep learning, object localization, disk herniation, disk bulge, image classification

## Abstract

**Background:**

Disk herniation and disk bulge are two common disorders of lumbar intervertebral disks (IVDs) that often result in numbness, pain in the lower limbs, and lower back pain. Magnetic resonance (MR) imaging is one of the most efficient techniques for detecting lumbar diseases and is widely used for making clinical diagnoses at hospitals. However, there is a lack of efficient tools for effectively interpreting massive amounts of MR images to meet the requirements of many radiologists.

**Objective:**

The aim of this study was to present an automatic system for diagnosing disk bulge and herniation that saves time and can effectively and significantly reduce the workload of radiologists.

**Methods:**

The diagnosis of lumbar vertebral disorders is highly dependent on medical images. Therefore, we chose the two most common diseases—disk bulge and herniation—as research subjects. This study is mainly about identifying the position of IVDs (lumbar vertebra [L] 1 to L2, L2-L3, L3-L4, L4-L5, and L5 to sacral vertebra [S] 1) by analyzing the geometrical relationship between sagittal and axial images and classifying axial lumbar disk MR images via deep convolutional neural networks.

**Results:**

This system involved 4 steps. In the first step, it automatically located vertebral bodies (including the L1, L2, L3, L4, L5, and S1) in sagittal images by using the faster region-based convolutional neural network, and our fourfold cross-validation showed 100% accuracy. In the second step, it spontaneously identified the corresponding disk in each axial lumbar disk MR image with 100% accuracy. In the third step, the accuracy for automatically locating the intervertebral disk region of interest in axial MR images was 100%. In the fourth step, the 3-class classification (normal disk, disk bulge, and disk herniation) accuracies for the L1-L2, L2-L3, L3-L4, L4-L5, and L5-S1 IVDs were 92.7%, 84.4%, 92.1%, 90.4%, and 84.2%, respectively.

**Conclusions:**

The automatic diagnosis system was successfully built, and it could classify images of normal disks, disk bulge, and disk herniation. This system provided a web-based test for interpreting lumbar disk MR images that could significantly improve diagnostic efficiency and standardized diagnosis reports. This system can also be used to detect other lumbar abnormalities and cervical spondylosis.

## Introduction

Magnetic resonance imaging (MRI) is a widely used technique for detecting lumbar disorders, and its advantages include high image quality and noninvasive and ionization-free radiation. Disk herniation and disk bulge are two common types of lumbar intervertebral disk (IVD) injuries that often result in low back pain and tingling and numbness in the legs [1,2]. The diagnosis of disk disorders is highly dependent on radiology methods such as MRI. The leading question is as follows: how can radiologists interpret massive amounts of magnetic resonance (MR) images quickly and accurately for real-world applications? Motivated by machine learning– and deep learning–based clinical practice [3-6], we propose an automatic diagnosis system for diagnosing disk bulge and disk herniation with MR images via deep convolutional neural networks (CNNs), which can reduce radiologists’ workload and provide the consistency required to produce standardized diagnosis reports.

Koh et al [7] proposed a computer-aided framework that uses several heterogeneous classifiers (ie, a perceptron classifier, a least mean squares classifier, a support vector machine classifier, and a k-means classifier) to construct a 2-level classification scheme for disk herniation diagnosis, which achieved 99% accuracy for 70 subjects. A probability classifier based on Gaussian models was proposed to detect abnormal IVDs. This model used the following three features: appearance, location, and context [8]. A study [9] on texture features that were obtained from IVD MR images used three different classifiers (ie, the back-propagation neural network, k-nearest neighbor, and support vector machine classifiers) to classify normal disks and IVDs and achieved a maximum accuracy of 83.33%. Additionally, many other methods have been proposed to automatically diagnose IVD diseases based on MR images [10-13]. Most of these models are for sagittal MR images, and there are very few studies that have used axial lumbar MR images, which are even more important in real clinical scenarios to identify disk bulge and herniation [13]. Most previous studies have mainly focused on binary classification (disease and normal) [7-9,11,12], as it is rare to study 2 diseases at the same time. In this study, we present a deep CNN–based diagnosis system for diagnosing lumbar disk bulge and disk herniation based on axial MR images. CNN analysis has proven to be an efficient method that is widely used to solve various image problems and has achieved huge success in many applicable fields [14-18].

This study aimed to develop a clinical applicable system that requires as little information from doctors as possible for diagnosing disk bulge and disk herniation via deep learning methods [19-21].

## Methods

### Data Set

**Figure 1:**
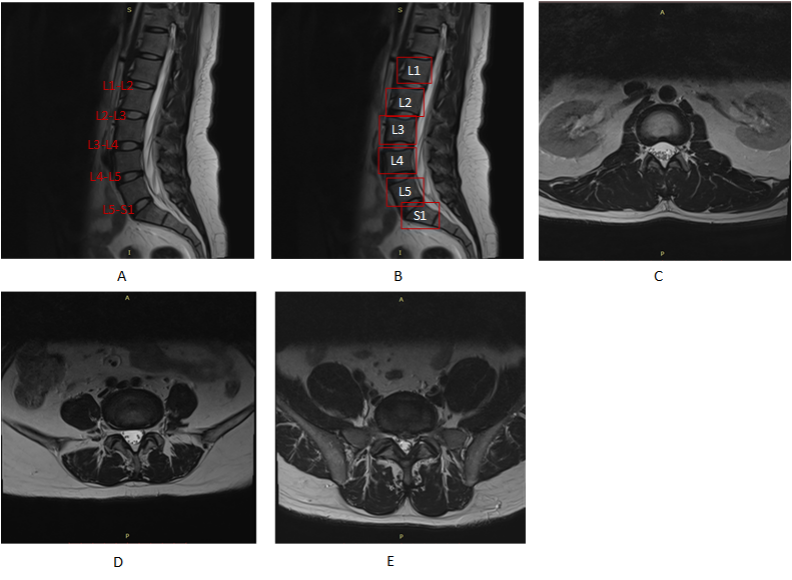
Examples of lumbar MR images. (A) A sagittal lumbar MR image in which 5 IVDs are labeled. (B) A sagittal lumbar MR image in which 6 vertebral bodies are enclosed in boxes. (C) An axial lumbar MR image of a normal disk. (D) An axial lumbar MR image of disk bulge. (E) An axial lumbar MR image of disk herniation. L: lumbar vertebra; MR: magnetic resonance; S: sacral.

In this study, lumbar MR Images and clinical diagnosis reports were collected from the Medical Imaging Department of Xi’an Number 3 Hospital, which is a large-scale grade 3A general hospital in Xi’an, China. The sagittal and axial T_2_-weighted lumbar MR images of 500 patients were acquired by using a Philips Ingenia 3.0T scanner and exported in the Digital Imaging and Communications in Medicine (DICOM) format. The main diagnosis was based on axial images, as they display the morphology of IVDs more clearly than other images. For each subject, midsagittal images were used to locate IVDs in axial images. A total of 3555 axial images were used in this study. These images were labeled as normal disk, disk bulge, and disk herniation according to diagnosis reports and rechecked by an experienced radiologist, as shown in Table 1. Examples of midsagittal lumbar images and axial images of normal disks, disk bulge, and disk herniation are shown in Figure 1.

**Table 1:** The number of axial images in each category.

Intervertebral disk	Normal images, n	Bulge images, n	Herniation images, n	Total, n
L1-L2^a^	593	37	36	666
L2-L3	549	120	30	699
L3-L4	347	284	86	717
L4-L5	158	413	178	749
L5-S1^b^	238	242	244	724
All intervertebral disks	1885	1096	574	3555

^a^L: lumber vertebra.

^b^S: sacral vertebra.

### Overall Diagnosis System

**Figure 2:**
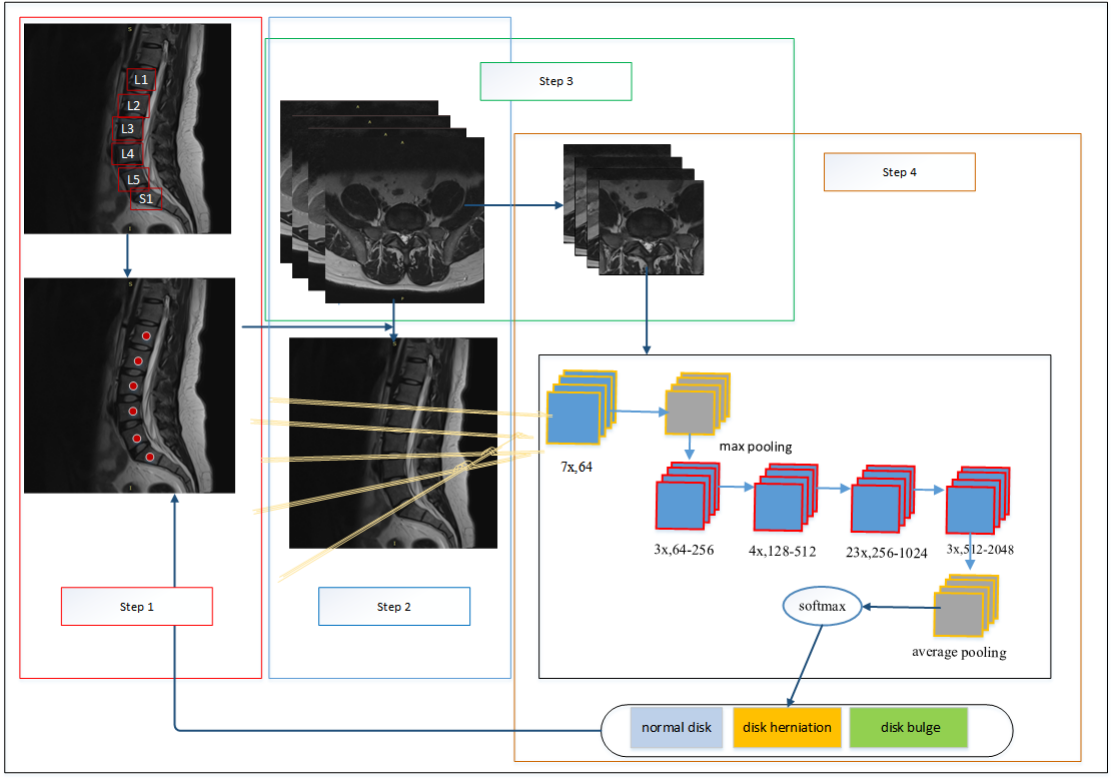
Overall diagnosis system. This system consists of 4 steps. First, vertebral bodies (L1, L2, L3, L4, and L5) in sagittal lumbar magnetic resonance images were automatically located by using the faster R-CNN, and the middle point of each vertebral body was calculated. Second, the axial images were grouped into 5 categories. Each category corresponded to an intervertebral disk (ie, the L1-L2, L2-L3, L3-L4, L4-L5, and L5-S1 intervertebral disks). Third, the intervertebral disk regions of interest in each axial MR image were segmented using the faster R-CNN. Finally, in each category, the region-of-interest images were classified as images of normal disks, disk bulge, and disk herniation using ResNet101. L: lumbar vertebra; R-CNN: region-based convolutional neural network; S: sacral.

Our system consists of 4 steps, as shown in Figure 2. In the first step, the six lumbar vertebral bodies (lumbar vertebra [L] 1, L2, L3, L4, L5, and sacral vertebra [S] 1) in midsagittal images were detected and located. The second step was to identify the corresponding IVDs in each axial MR image. Afterward, these axial images were grouped into five categories (L1-L2, L2-L3, L3-L4, L4-L5, and L5-S1). In the third step, the IVD regions of interest (ROIs) in axial images were segmented to decrease the noise of the images. In the fourth step, each ROI image that included the five IVDs was classified as normal disk, disk bulge, or disk herniation.

### Automatically Locating Vertebral Bodies in Midsagittal Images

The faster region-based CNN (R-CNN) [19] was developed from the R-CNN [22] and the fast R-CNN [23], which unifies the target detection process (including candidate region generation, feature extraction, classification, and position refinement) into 1 deep network framework and greatly improves operational speed. In step 1, the faster R-CNN was used to locate the vertebral bodies in sagittal MR images.

First, the six vertebral bodies (L1-S1) in 200 midsagittal images were manually located under the guidance of a radiologist. Second, the faster R-CNN was trained to detect and locate each vertebral body. We detected vertebral bodies instead of disks because they were easier to manually locate. Finally, the middle point coordinate of each vertebral body was calculated based on bounding box coordinates, as the precise location of the vertebral bodies would be used to locate the vertebrae in axial MR images, as shown in Figure 1 (step 1).

The faster R-CNN was implemented with Caffe [24] (Berkeley Vision and Learning Center deep learning framework) and trained in parallel on 4 Nvidia Titan X graphics processing units. Accuracy, sensitivity, and specificity [25,26] were analyzed to comprehensively evaluate the performance of this system.

### Identifying the Corresponding IVD in Each Axial MR Image

**Figure 3:**
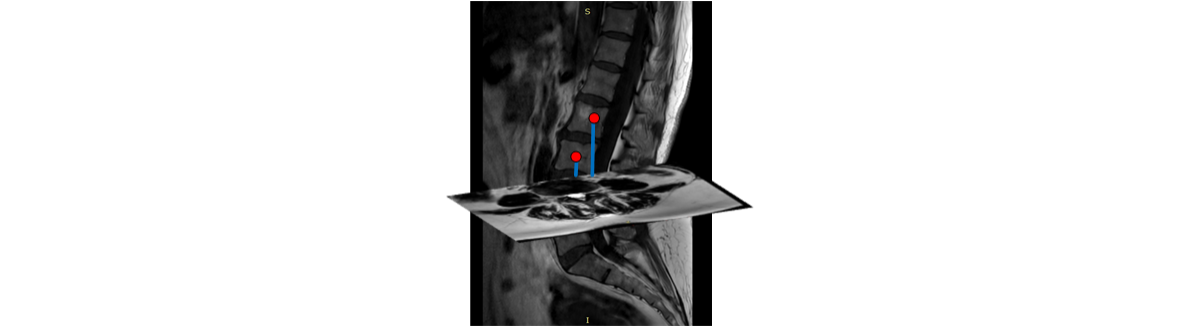
The intervertebral disks (from L1-L2 to L5-S1) in each axial image were located by calculating directed distances. The red dot shows the middle point of each vertebral body in a sagittal image. The blue line depicts the directed distance from the red dot to a specific axial image. L: lumbar vertebra; S: sacral.

For each subject, 15 axial slices were needed to identify the corresponding IVDs (L1-L2, L2-L3, L3-L4, L4-L5, and L5-S1) in each axial MR image. In step 1, the center point coordinates of the six vertebral bodies in the sagittal images were calculated. The directed distances from these center points to each axial image were calculated for each subject based on the spatial location relationship between sagittal images and axial images. The directed distances indicated which IVDs were closer to the corresponding IVDs in each axial image and which IVDs were located above or below the corresponding IVDs, as shown in Figure 3. Based on these distances, the axial slices were classified into 5 categories (L1-L2, L2-L3, L3-L4, L4-L5, and L5-S1). The conversion from DICOM patient-based coordinates to 2D computer coordinates was conducted in order to establish the relationship between the primitively processed images and the 3D DICOM coordinates. The detailed procedures are depicted in Multimedia Appendix 1.

### Locating IVD ROIs in Axial MR Images

Axial lumbar MR images contain large amounts of unrelated areas. In order to focus on IVDs and extract more relevant features, IVD areas were labeled manually in 1237 axial images, including normal disk areas, bulging disk areas, disk herniation areas, and the L1-L2 to L5-S1 IVD areas. The IVD areas of each ROI image needed to be located to train the faster R-CNN, and our fourfold cross-validation showed 100% accuracy. Afterward, the ROIs in each axial lumbar image were detected and extracted using the faster R-CNN, as shown in Figure 2 (step 3). We reserved a larger area for the components surrounding IVDs, as they may also help with identifying the condition of the disks (eg, the compression of the spinal canal).

### Classification of ROI Images

It is worth mentioning that the degradation problem of the ultradeep CNN may result in reduced classification accuracy as the depth of the CNN increases. He et al [27] proposed a deep residual network framework that can solve this problem by using the residual block method, and this was proven to have significant accuracy for the ImageNet validation set [27-29]. The residual architecture of ResNet101 is shown in Figure 2 (step 4).

According to the diagnosis reports, in every category (L1-L2 to L5-S1), a total of 3555 axial MR images were labeled as normal disk, disk bulge, or disk herniation. All 3555 ROI images were reviewed by an expert radiologist to confirm whether the images conformed to the labels. Afterward, ResNet101 was used to conduct the 3-class classification for each category, and our fourfold cross-validation showed classification accuracies of 92.7%, 84.4%, 92.1%, 90.4% and 84.2% for the L1-L2, L2-L3, L3-L4, L4-L5, and L5-S1 IVDs, respectively. In this step, a cost-sensitive CNN was used to test for imbalances in the 3-class classification data set [30]. Relevant mathematical theory is provided in Multimedia Appendix 1.

## Results

We focused on images that showed disk bulge, disk herniation, and normal disks. From Table 1, we can see that the probabilities of disk bulge and disk herniation in the L1-L2 and L2-L3 IVDs are low, and disk bulge tended to occur more commonly in the L3-L4, L4-L5, and L5-S1 IVDs. The L5-S1 IVD is the most common location of disk herniation. This is probably because it bears more weight and pressure than the other locations.

## Discussion

### Principal Findings

**Figure 4:**
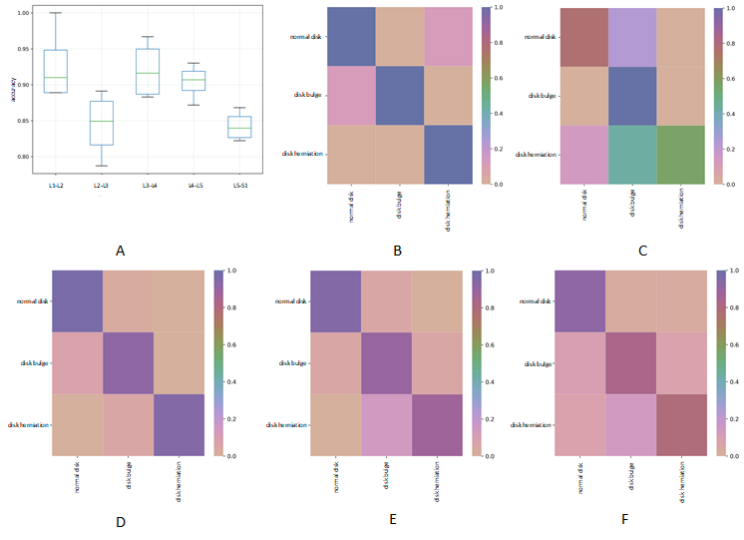
Results of the 3-class classification (normal disk, disk bulge, and disk herniation). (A) The average accuracies of the classification system (calculated using ResNet101) for the following five categories: L1-L2, L2-L3, L3-L4, L4-L5 and L5-S1. The rows and columns of all heat maps represent ground truth labels and predicted labels, respectively. The x-axis shows the five intervertebral disks. (B) A heat map of the classification accuracies for category L1-L2. The color scale expresses the accuracy. (C) A heat map of the classification accuracies for category L2-L3. The color scale expresses the accuracy. (D) A heat map of the classification accuracies for category L3-L4. The color scale expresses the accuracy. (E) A heat map of the classification accuracies for category L4-L5. The color scale expresses the accuracy. (F) A heat map of the classification accuracies for category L5-S1. The color scale expresses the accuracy. L: lumbar vertebra; S: sacral.

Our system is comprised of 4 steps. First, the system automatically located vertebral bodies (from L1 to S1) in sagittal images by using the faster R-CNN, which was trained on 200 manually cropped images. Our fourfold cross-validations showed 100% accuracy. This high location accuracy shows that the faster R-CNN method can more accurately locate vertebral bodies than many other methods, such as the Gabor filter bank method [31], which is a method based on measurements of disk signal intensity and structure [7]. Second, the disk positions (from L1-L2 to L5-S1) in each axial image were calculated based on the equations for coordinate conversion. We achieved an accuracy of 100%. Third, the system automatically segmented IVD ROIs in axial MR images by using the faster R-CNN, which was trained on 1300 manually boxed images that included all five types of disks (from L1-L2 to L5-S1) and the disk conditions (normal, herniation, and bulge). The mean average precision [21] reached 100%. This high accuracy was the result of the excellent performance of the faster R-CNN. Finally, all ROI images were classified as normal, bulge, and herniation by using ResNet101. The average accuracies for the 3-class classification of the L1-L2, L2-L3, L3-L4, L4-L5, and L5-S1 IVDs were 92.7%, 84.4%, 92.1%, 90.4%, and 84.2%, respectively. All relevant results are shown in Figure 4. Previous studies have mainly focused on comparing IVDs affected by 1 disease (disk bulge or herniation) with normal IVDs. This is known as a binary classification. For example, the performance value of one IVD classification system was 86.5%, and this was based on a sparse shape reconstruction from a statistical shape model [32]. Additionally, an accuracy of 92.78% was reported by a study that classified normal disks and disk bulge by using a program called IVD Descriptor [13]. Compared to the accuracies of these previous studies, our accuracies were roughly the same or slightly inferior. This was mainly because a 3-class classification system is often less accurate than a binary classification system.

Based on our results, the classification accuracies for the L2-L3 and L5-S1 IVDs were lower than those for other disks. The shape of a normal disk is somewhat different from the L1-L2 to L5-S1 IVDs. With regard to the L2-L3 disks, several images were blurry, and it was difficult to identify subtle differences. This, coupled with our small sample of herniated disks, had a considerable impact on our classification accuracy. Data quality may become a crucial factor that could restrict the performance of algorithms used in research [33]. With regard to the L5-S1 disks, the normal disks were similar in shape to that of bulged disks in axial images. There were also a few images that were wrongfully classified by our system, which resulted in a lower classification accuracy.

**Web-Based Diagnosis System**

**Figure 5:**
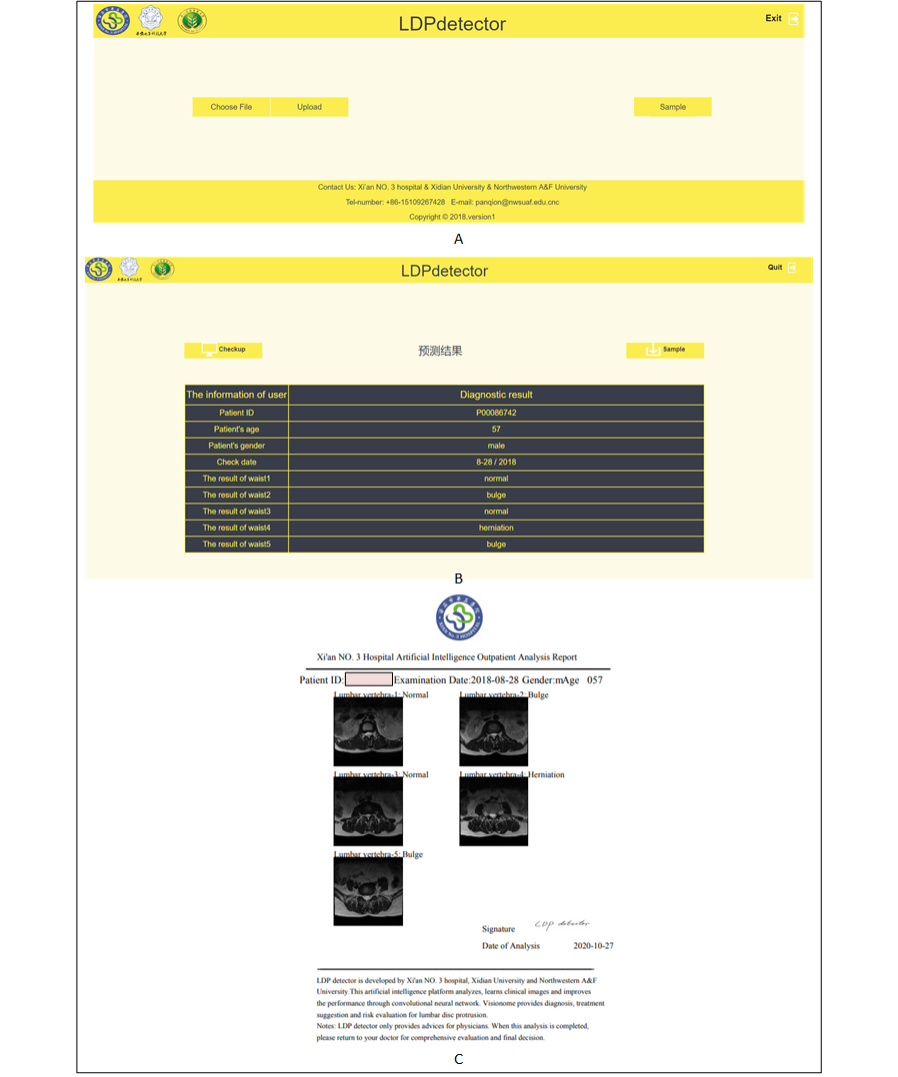
Appearance and functions of the reports of the web-based automatic diagnostic system. (A) This is the page for uploading a folder. (B) Diagnostic results in tabular form. (C) The diagnostic report in the Unified format. LDP: lumbar disk protrusion.

We used the Django framework [34] to develop an automatic diagnosis system for radiologists that could analyze inputted medical images and show results as normalized diagnosis reports (a PDF file). The appearance and functions of the reports are shown in Figure 5. This system can be deployed in multiple radiology departments to analyze patients’ lumbar MR images and collect more images to improve radiologists’ IVD interpretation performance. This system is freely available [35].

In this paper, we present an automatic diagnosis system for diagnosing disk bulge and disk herniation with axial MR images via deep convolutional neural networks. This system can automatically determine the position and the condition of IVDs in axial MR images. Therefore, this system could help reduce the workloads of radiologists by analyzing lumbar MR images via a standardized method. In addition, this system can be expanded to analyze other types of lumbar diseases, such as cervical spondylosis. However, there are some limitations to using this system. Data from this system could be fundamentally limited by the quality of images (eg, when the image is blurry), making it difficult to identify subtle differences. The system is also limited by the size of the total data set, as it is relatively small for deep convolutional neural networks. Our future work will focus on the following two aspects: (1) developing this system by using a more targeted method that analyzes the specific features of MR images, and (2) gathering more MR images to train a more practical and complete automatic diagnosis system.

## Appendix

Multimedia Appendix 1 Supplementary materials.

## Abbreviations

CNN: convolutional neural network
DICOM: Digital Imaging and Communications in Medicine
IVD: intervertebral disk
L: lumbar vertebra
MR: magnetic resonance
MRI: magnetic resonance imaging
R-CNN: region-based convolutional neural network
ROI: region of interest
S: sacral vertebra
