# Supplementary material for: Automatically Diagnosing Disk Bulge and Disk Herniation With Lumbar Magnetic Resonance Images by Using Deep Convolutional Neural Networks: Method Development Study
Source: JMIR Med Inform. 2021 May 21;9(5):e14755. doi: 10.2196/14755 (PMC8178733; doi:10.2196/14755)
Supplement: Multimedia Appendix 1 [file medinform_v9i5e14755_app1.docx]

**Part 1**

First, we need to establish relations between standard DICOM patient-based coordinate system and pixel coordinate system. The DICOM file provides a description of the direction cosines for the image through the Image Orientation tag (0020, 0037) as equation (1) in Supplementary Materials

$v_{r}={(v_{r1},v_{r2},v_{r3})}^{T}, v_{c}={(v_{c1},v_{c2},v_{c3})}^{T}$ (1)

where, $v_{r}$,$v_{c}$ are the directions of the first row and first column of the image in patient-based coordinate system, superscript T represents the transpose of a vector. Image Position tag (0020, 0032) is the coordinates of the upper left-hand corner of the image as a vector

$P_{0}={(x_{0},y_{0},z_{0})}^{T}$ (2)

Pixel Spacing (PS) tag (0028, 0030) is the Physical distance in the patient between the centers of each pixel. Suppose P is one point of the image, its coordinate is ${(x_{1},y_{1},z_{1})}^{T}$ in patient-based coordinate system and its pixel coordinate is${(x,y)}^{T}$, the equation of coordinate transformation is as follows.

$\left[ \begin{matrix} x_{1} \\ y_{1} \\ z_{1} \end{matrix} \right]=\left[ \begin{matrix} v_{r1} & v_{c1} & x_{0} \\ v_{r2} & v_{c2} & y_{0} \\ v_{r3} & v_{c3} & z_{0} \end{matrix} \right]\left[ \begin{matrix} x \\ y \\ \frac{1}{PS} \end{matrix} \right]*PS$ (3)

Then, foot points of each middle point of vertebral bodies (MPIB) to each axial MR image were determined, we got some vectors which starting from MPIBs and ending at foot points. Supposed point M is one of middle point of vertebral bodies and its coordinate is ${(x_{m1},y_{m2},z_{m3})}^{T}$，the foot point of M in a axial MR image was denoted as $F={(x_{f1},y_{f2},z_{f3})}^{T}$.

$\left\{ \begin{aligned} (MF,v_{r})=0 \\ (MF,v_{c})=0 \\ (MF,P_{0}F)=0 \end{aligned} \right.$ (4)

($MF,v_{r}$) is the inner product of vector $MF$ and$v_{r}$. Equations (4) can help us to calculate the coordinate or the approximate coordinates of foot point F. The orientation and length of vector $MF$ will determine the corresponding intervertebral disc for each axial MR image, as shown in Fig.3.

**Part 2**

Given$\left\{ \left( x_{1},y_{1} \right),\left( x_{2},y_{2} \right),\cdots,\left( x_{l},y_{l} \right) \right\},x_{i}\in R^{n},y_{i}\in\{-1，1\}$, the loss function of the cost sensitive CNN is shown as following,

$L\left( w \right)=-\frac{1}{m}[\sum_{i=1}^{m} \sum_{j=1}^{k} Class_{-}weight\left\{ y_{i}=j \right\}*log\frac{e^{w_{j}^{T}x_{i}}}{\sum_{s=1}^{k} e^{w_{s}^{T}x_{i}}}]+\frac{\lambda}{2}\sum_{i=1}^{k} \sum_{j=1}^{n} w_{ij}^{2}$ (5)

where, $m,w,n and k$ represent the size of mini-batch, the parameter, the number of input neurons and the number of classes respectively. $Class\_weight\left\{ y_{i}=j \right\}$ is the weight of a sample in class j and $\frac{\lambda}{2}\sum_{i=1}^{k} \sum_{j=1}^{n} w_{ij}^{2}$ is the penalization term.
